# Animal Models of Autistic-like Behavior in Rodents: A Scoping Review and Call for a Comprehensive Scoring System

**DOI:** 10.3390/ijms251910469

**Published:** 2024-09-28

**Authors:** Asher Ornoy, Boniface Echefu, Maria Becker

**Affiliations:** 1 Department of Morphological Sciences and Teratology, Adelson School of Medicine, Ariel University, Ariel 40700, Israel; bonifacee@ariel.ac.il (B.E.); mariabe@ariel.ac.il (M.B.); 2 Hadassah Academic College, Jerusalem 9101001, Israel; 3 Hadassah Medical School, Hebrew University, Jerusalem 9112102, Israel

**Keywords:** ASD-like behavior, animal models, neurodevelopmental disorders, scoring system

## Abstract

Appropriate animal models of human diseases are a cornerstone in the advancement of science and medicine. To create animal models of neuropsychiatric and neurobehavioral diseases such as autism spectrum disorder (ASD) necessitates the development of sufficient neurobehavioral measuring tools to translate human behavior to expected measurable behavioral features in animals. If possible, the severity of the symptoms should also be assessed. Indeed, at least in rodents, adequate neurobehavioral and neurological tests have been developed. Since ASD is characterized by a number of specific behavioral trends with significant severity, animal models of autistic-like behavior have to demonstrate the specific characteristic features, namely impaired social interactions, communication deficits, and restricted, repetitive behavioral patterns, with association to several additional impairments such as somatosensory, motor, and memory impairments. Thus, an appropriate model must show behavioral impairment of a minimal number of neurobehavioral characteristics using an adequate number of behavioral tests. The proper animal models enable the study of ASD-like-behavior from the etiologic, pathogenetic, and therapeutic aspects. From the etiologic aspects, models have been developed by the use of immunogenic substances like polyinosinic-polycytidylic acid (PolyIC), lipopolysaccharide (LPS), and propionic acid, or other well-documented immunogens or pathogens, like *Mycobacterium tuberculosis.* Another approach is the use of chemicals like valproic acid, polychlorinated biphenyls (PCBs), organophosphate pesticides like chlorpyrifos (CPF), and others. These substances were administered either prenatally, generally after the period of major organogenesis, or, especially in rodents, during early postnatal life. In addition, using modern genetic manipulation methods, genetic models have been created of almost all human genetic diseases that are manifested by autistic-like behavior (i.e., fragile X, Rett syndrome, SHANK gene mutation, neuroligin genes, and others). Ideally, we should not only evaluate the different behavioral modes affected by the ASD-like behavior, but also assess the severity of the behavioral deviations by an appropriate scoring system, as applied to humans. We therefore propose a scoring system for improved assessment of ASD-like behavior in animal models.

## 1. Introduction

Animal modeling of human diseases significantly contributes to the enhancement of our comprehension of disease etiology, pathogenesis, and potential treatments [1,2,3]. Various mammalian models, including mice, rats, rabbits, pigs, and non-human primates, have been developed due to genetic proximity to humans, easy handling, and relevance to specific disease aspects [3]. In the last several decades, non-mammalian animals have also been used, including amphibians, avian models, and, lately, zebrafish [3,4,5,6,7]. Generally, animal models for human diseases are required to meet three basic values: face validity, when animals recapitulate disease phenotype in similarity to humans; etiologic (construct) validity (relevance), when pathophysiological processes in animals are similar to those that cause disease in humans; and predictive validity (pharmacologic sensitivity), when animals respond to medications that are effective to treat the human disease [8]. Often the causation of the human diseases and of the disease in animal models are similar, as are the symptoms, complications, and treatment. Hence, there are genetic and non-genetic animal models used for the study of almost all human diseases.

The American Psychiatric Association’s Diagnostic and Statistical Manual, Fifth Edition, 2013 (DSM-5) provides standardized criteria to diagnose ASD [9]. The diagnostic features associated with ASD are a triad of impaired social interactions, verbal and nonverbal communication deficits, and restricted, repetitive behavioral patterns that may also be associated with somatosensory and special senses impairments.

Careful phenotypic characterization of ASD animal models is essential to ensure that they accurately recapitulate key features of the human disorder. This includes assessing behavioral, cognitive, social, and communication deficits that are relevant to human symptoms. If there are only a few behavioral changes, or behavioral tests have not been sufficiently applied to assess most of the typical ASD-like behaviors, the resemblance to human ASD is incomplete.

Modeling in animals of neurodevelopmental disorders such as ASD is challenging and complex because the etiology and pathogenesis of ASD are multifactorial and still unclear [10]. One significant difficulty is that ASD is presently diagnosed based on a set of core behavioral abnormalities rather than objective biomarkers [11]. The diagnostic criteria for ASD rely on observable behaviors such as impaired social interaction, communication deficits, and repetitive or restricted interests and behaviors. Unlike disorders with clear physiological markers, such as certain genetic conditions or infectious diseases, ASD lacks physiological biomarkers that can be easily measured or quantified. Furthermore, the heterogeneity of ASD etiology encloses a wide range of symptoms and a wide range of severity levels [10]. Therefore, the development of animal models that may reflect this variability of ASD is difficult, and requires accounting for genetic diversity and environmental and developmental factors that contribute to the disorder. Many currently established ASD-like animal models, either induced or genetic, exhibit behavioral traits associated with ASD. However, many models still fail to fully reflect the complexity of human ASD.

While there are no established biological markers for ASD, identifying translational biomarkers in animal models can facilitate the translation of preclinical findings to clinical settings. This may include molecular, neuroimaging, or electrophysiological biomarkers that reflect underlying neural circuitry abnormalities or treatment responses.

Several strategies are utilized for designing mouse models of ASD [12,13]. Genetic models using knockout techniques, Transcription Activator-Like Effector Nucleases (TALENs) and CRISPR/Cas9, and gene editing technologies introducing mutations or deletions in genes associated with ASD in mice can mimic genetic factors contributing to the disorder in humans. This includes manipulating genes such as Methyl-CpG Binding Protein 2 (MeCP2), fragile X messenger ribonucleoprotein 1 (FMR1), SH3 and Multiple Ankyrin Repeat Domains 3 (SHANK3), and neuroligin (NLGN), which have been implicated in ASD, often with clinical differences between genders [14].

Many tests are employed in animals to assess various behavioral deviations. The utilization of appropriate behavioral tests for each core behavior is crucial for establishing conviction for the validity of the core domain of the autism symptoms being assessed. The domains, based on the criteria that define autistic subjects in *DSM-5*, are social interaction, communication and motivation, and restrictive-repetitive behaviors, as well as tests for the assessment of various behavioral changes often observed in autism like anxiety, learning and memory difficulties, and sensory and motor difficulties.

We will present various studies describing different animal models of ASD-like behavior that used different behavioral tests diagnostic for specific neurobehavioral features found in ASD. Generally, investigators did not define the severity of these behavioral deviations and sometimes assessed only a few of the typical autistic-like behaviors. This raises the question in which conditions are the models only demonstrating features of autistic-like behavior that may sometimes be found in various neuropsychiatric disorders or demonstrating the full spectrum of autistic-like behavior. An additional question is whether a scoring system, similar to that used in human, will improve the face and construct validity of these models.

Behavioral categorization may guide researchers to the development and implementation of comprehensive and acceptable scoring methods for validating animal models of ASD-like phenotypes. It is noteworthy that ASD is diagnosed only if there is a set of well-defined clinical presentations. Hence, to prove that the animal exhibits ASD-like behaviors, studies must demonstrate characteristic behavioral features that can be delineated only when using a variety of behavioral tests. Generally, relying on too few behavioral tests demonstrates only specific behavioral deviations that may be representative of ASD but also of other neuropsychiatric disorders as they demonstrate only some of the characteristic features of ASD. We are not suggesting, however, that a single model must show all features of ASD, but rather advocate for a reproducible behavioral phenotyping that is robust enough for predictive validity.

In this review we will discuss genetic and non-genetic models of ASD, the way they are produced and the methods for verification that the clinical behavioral symptoms are like those observed in human ASD. We will also discuss briefly various tests for the assessment of behavioral deviations associated with ASD and propose a scoring system to assess the severity of the ASD-like behavioral symptoms.

## 2. Brief Description of Behavioral Assays That Define ASD-like Behaviors in Rodents

There are many tests for the evaluation of the different behaviors in animals, especially in rodents. Moreover, several specific tests often measure the same behaviors. We will not discuss these different behaviors at length, but describe them only briefly according to the behavioral features they are meant to measure.

**Figure 1:**
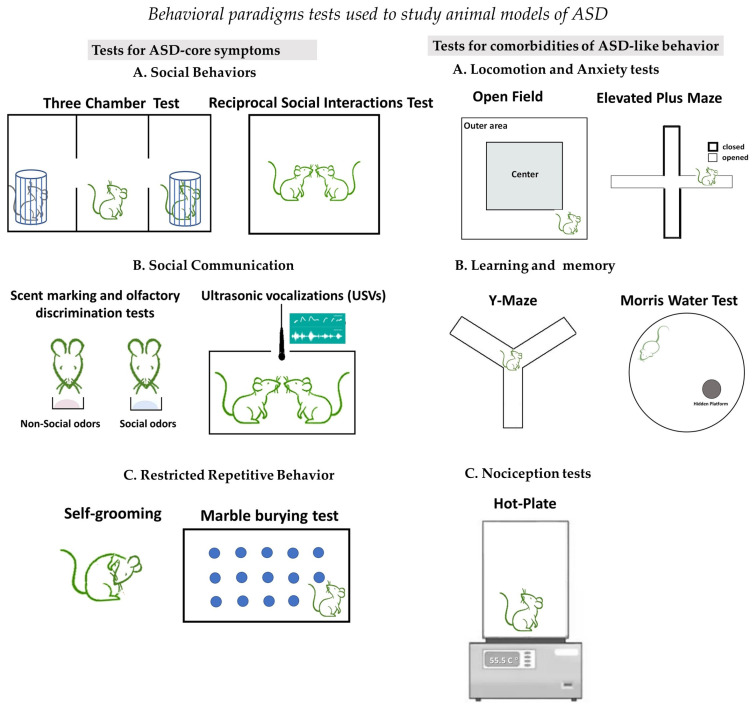
Behavioral tests used to study animal models of ASD. The diagrams present graphical illustration of mostly used behavioral tests for evaluation of various aspects of ASD-like behaviors. Tests for ASD-core symptoms are included in the left side of the figure. (**A**) Three-chamber and reciprocal social interaction tests are used for assessment of paradigm of social interest and social memory. (**B**) Scent marking and olfactory discrimination tests are used for discrimination of social cues based on odor olfaction. (**C**) Self-grooming and marble burying tests are used for assessment of restricted repetitive movements. Tests for comorbidity that may accompany the main ASD-behaviors are included in the right side of the Figure. (**A**) Open field is mainly used to assess locomotion, motivation and anxiety levels, whereas Elevated Plus Maze are used to assess anxiety level. (**B**) Y-Maze and Morris water maze are used for evaluation of learning and memory impairments. (**C**) Hot-plate is used to assess the nociception threshold of sensory perception.

These behavioral tests are grouped into batteries and described briefly with reference to studies describing them in detail. These test batteries are namely for social interaction, communication, restrictive-repetitive behaviors, and comorbidities ( Figure 1 ).

### 2.1. Tests to Investigate Social Behaviors

The three-chamber test for sociability and novel social preference, originally developed by Crawley and colleagues, is the most widely used measure for sociability and social recognition assays in rodents [13,15]. The objective of test initially to test for social interaction between a subject and conspecific can now be expanded to concomitantly test also for social preference [16,17,18,19,20,21]. The social partition test is similarly employed to assess abnormality in social behavior of ASD-like rodent models [22]. The reciprocal social interactions assay is used to observe how animal reciprocates social advances (consisting of sniffing, following, chasing, pushing past, crawling over, pushing under, grooming, fast-paced wrestling, pouncing, pinning, chasing, and boxing) made towards it by a conspecific [13,23]. The test for social motivation is primarily used to evaluate how rewarding a test animal finds a socially conditioned environment [13]. With proper application of these standardized tests in various contexts and results comparison, researchers can gain a more comprehensive understanding of the subject’s social behavior that may be cryptic in one test only. Corroboratory assays may show social behavior that can differ across different settings, helping to interpret the findings in the presence of potential physical or cognitive deficit. The overall goal of these tests is to provide a reliable and systematic way to characterize social behaviors in animal models, which can contribute to the understanding of the underlying mechanisms and development of potential interventions for social impairments observed in ASD.

### 2.2. Tests to Investigate Social Communication

The scent marking test aims to investigate non-verbal communication in rodents. Naturally, rodents communicate via olfactory signals such as scent markings and pheromones [24]. The paradigm can be modified for habituation and dishabituation (OHDH) test [25,26]. The latter paradigm serves the same purpose as the olfactory discrimination index test [27]. Ultrasonic vocalizations (USVs) are the major communication sounds used by rodents at reported frequencies ranging between 30 to 110 kHz [28,29,30]. USV emissions can provide evidence of an early-life social communication abnormality [29,31,32,33] and can be a tool for early behavioral ASD phenotyping [33] 

Olfactory signals and ultrasonic vocalizations provide complementary approaches to study communication in rodent models of ASD. Test results provide valuable insights into the social communication deficits present in ASD-like phenotypes for a better understanding of the underlying mechanisms, and the development of early detection and intervention strategies.

### 2.3. Tests to Investigate Restricted Repetitive Behaviors

Restricted, repetitive patterns of behavior and interests in ASD can include any stereotyped or repetitive motor movements, insistence on sameness and inflexible routines, highly restricted and fixated interests, hyper/hypo-reactivity to sensory input, and unusual fascinations and fixation on objects [9,34]. It is therefore practically impossible to have one paradigm assay that can sufficiently test these behavioral patterns. This highlights again the need to evaluate more than a single task for several domains of ASD when designing animal models.

Below are some of the most widely employed techniques that can be used to observe some repetitive patterns in rodent models of autistic-like behavior.

The marble burying test examines in rodents the presence of obsessive/compulsive repetitive digging behavior, a typical autistic-like model [35]. Repetitive behaviors in rodents can also be observed in normal home cage activities like self-grooming, bedding, backflipping, circling, and jumping [36,37]. Other tests, for restricted repetitive interest include novel object/hole, the Y-Maze [13], the water T-maze (WTM), and the Barnes maze [38,39].

### 2.4. Tests to Investigate for Comorbidities of ASD-like Behaviors

Below are snippets of few corroboratory tests that can be employed for comprehensive assessment across cognitive, motor, and sensory domains to fully characterize the ASD phenotype and avoid potential confounds.

The Y-Maze Spontaneous Alternation test measures hippocampal function and short-term memory [20,40]. The novel object recognition (NOR) test assesses intellectual difficulties, which affect around 70% of ASD cases [13]. The Morris water maze (MWM) tests spatial learning, memory, and reversal learning [41]. The fear conditioning test measures fear memory by associating a foot shock with contextual or auditory cues [42] Motor coordination and balance paradigms, e.g., the rotarod and beam walking, assess motor coordination, balance, and motor skill learning [43]. Anxiety-like behavior is assayed using an elevated plus maze and light–dark box tests [39,44]. The open field locomotory test assesses general locomotion and exploratory behavior [16,36]. Nociception tests are performed using hot-plate and tail-flick tests measure sensory processing [45,46], with ASD models sometimes showing enhanced nociception or hyperalgesia [47,48].

## 3. Non-Genetic Models of ASD-like Behaviors

Non-genetic rodent models are widely used models of ASD-like behavior because of their preclinical and clinical relevance, validity in disease etiology, and resemblance to human symptoms [5,49]. They are manipulated to mimic environmentally induced autistic-like behavior in humans. Rodents are prenatally or early postnatally exposed to a variety of chemical substances or biological maneuver to demonstrate the validity of the use of animal models to recapitulate ASD-like phenomena and symptoms [39,40]. These models have continued to advance the understanding of the different key behavioral and neurobiological features observed in human ASD, namely asocial and repetitive behaviors, communication deficits, and cognitive impairment. These studies collectively validate important mechanisms involving genetic and epigenetic factors, immune dysregulation, oxidative stress, and environmental constraints in the development and progression of ASD. These models have revealed vital information on potential targets for further research and therapeutic interventions. The more recent ones will be discussed briefly.

### 3.1. Biological Models of ASD-like Behaviors

Maternal immune activation (MIA), or activation of the maternal immune system by inflammation processes, is thought to play a significant role in the development of autism [39,50,51,52]. Several rodent models of ASD-like behavior have been developed to recapitulate human disease phenotype by eliciting infectious processes prenatally or early postnatally using varieties of biological maneuvers [53,54]. A nonhuman primate model (rhesus monkey) administered maternal immunoglobulin G (IgG) class antibodies that were purified from mothers of ASD children produced offspring with ASD-like behavior [55]. Using a three-chamber approach, the IgG-ASD-like offspring was asocial to conspecific and showed impaired reciprocal social interaction. Magnetic resonance imaging of the brain revealed that male IgG-ASD offspring had enlarged frontal lobes white matter volume compared with controls [55]. Jones et al. and Bruce et al. generated mouse and rat models of maternal immune activation (MIA) [44,53] by injecting females with multiple antigenic epitopes two weeks before pregnancy to activate specific autoantibody production. Following pregnancy and parturition, pups were tested for neurodevelopmental milestones from PND 4-14. The adults were assessed for behavioral deficits using the battery of behavioral assays: reciprocal social interaction, three-chamber approach, repetitive grooming, marble burying, USVs, Morris water maze, open field, light and dark exploration, and elevated plus maze. Results in mice showed no difference between the offspring of MIA and control in the three-chamber social approach, although there was significant repetitive self-grooming, reduced vocalization to social cues, and altered neurodevelopmental trajectory in pups. In rats, the pups showed lower body temperature, reduced pup calls, and increased performance in negative geotaxis. Exposed adults showed significantly less interest in social interaction [44,53]. Most frequently, polyinosinic-polycytidylic acid (PolyIC), a double-stranded RNA molecule that stimulates an immune response through the activation of toll-like receptor 3, has been widely employed to model autistic-like behavior in rodents [56,57,58] (Table 1).

Lipopolysaccharide (LPS) and propionic acid are also well-documented immunogens used pre- and postnatally to produce rodent models of ASD-like behavior [59,60,61,62]. Wu et al. induced MIA in zebrafish 24 h before mating by treating females with Poly(I:C) intraperitoneally [61]. Their findings from behavioral assays—three-chamber, shoaling, open field, and social preference test—suggest that offspring of MIA induced mothers exhibited impaired social approach and social cohesion, similar to human ASD phenotypes. These findings were attributed to mediation by toll-like receptors 3 and 4, and the role of villin-1 (vil1) pathway. In recent past, pathogens like *Mycobacterium tuberculosis*, have also been reported as inducing factors for ASD [63]. These modeling methods are achieved by the maternal response to induced infection resulting in immunological dysregulation, or by activation of the immune system in the absence of infection that could cross the placenta [65].

These models of autistic-like behavior produced by maternal immune activation provide important insights on significant risk factor for ASD. They enable investigation of the underlying biological mechanisms and translational research to advance human autism diagnostics and management. The models hold potential that may aid biomarkers identification for early detection of autism risk in children. This study continues to shed light on the complex etiology of autism and encourages rigor in future research strategies.

### 3.2. Chemical Models of ASD-like Behaviors

Several chemical compounds have been used to demonstrate environmental components in the etiology of ASD-like phenotype in humans and animals. Epidemiologic studies have shown that there is 3–5% increase rate of ASD among offspring of epileptic mothers, who at the time of pregnancy undertake valproic acid (VPA) treatment, compared to the general population [66]. Exposing pregnant rodents to VPA has been established as a modeling tool for studying ASD-like behavior [49,66,67]. VPA models have demonstrated both construct and face validity in similarity to human ASD symptoms [14,49]. During pregnancy and early post-natal life, exposure to VPA is associated with impaired behaviors as manifested by different behavioral tests [39]. Interestingly, some VPA rodent models show a sex-dependent variation in behavioral outcome as commonly seen in humans with autism [68]. In addition to behavioral deficits, a reduced number of neurons in motor cranial nerves nuclei, reduced Purkinje cells, and size of the cerebellar hemispheres, as well as changes in the expression of many genes, some of them found to be associated with human ASD, have been reported in VPA-induced ASD-like behavioral models [49] (Table 2).

We produced an ASD-like phenotype in ICR outbred mice with 300 mg of VPA on PND 4. ICR mice are widely used outbred strains, known for their more genetic variation background as compared to inbred strains, mimicking genetic diversity in the human population [74,75]. Starting from PND 50, mice were assessed on three-chamber, elevated plus, water T-maze, and open field test. VPA models exhibited neurobehavioral deficits typical of ASD that were more prominent in males. Altered expression of antioxidant genes in the prefrontal cortex and enhanced oxidative stress were observed [40]. The observed ASD-like behavior must have resulted from epigenetic changes. Further studies revealed changes in the expression of 146 neuropathology and neurophysiology genes, some of them known to be involved in the neuropathology of ASD [76].

To study the involvement of purinergic signaling system on the development of ASD, a VPA rat model of ASD was generated by treating pregnant Wistar rats with 600 mg/kg of VPA on GD 12.5 [37]. Behavioral studies using three-chamber, reciprocal social interaction, open field/self-grooming and elevated plus maze paradigm showed that VPA induced in the offspring decreased reciprocal social interactions, impaired sociability index, and increased anxiety and nociceptive threshold. They also showed that VPA induced upregulation of interleukin 6 (IL-6), P2X4, and P2Y2 receptor expression in the hippocampus and medial prefrontal cortex [64]. 

Chen et al. performed ASD-related assays on VPA-treated zebrafish at different developmental time points [69]. They revealed that VPA induced a hyperactive movement disorder and increased time spent in the light area with less crossing in zebrafish. There was significantly less attack in the mirror test and time spent in the mirror zone. They found increased distance between VPA offspring in the shoaling test, and less contact duration and frequency when compared to the controls. There was an increase in cell and neural stem cell proliferation in the brain region, which might have contributed to the brain overgrowth macrocephaly observed [69].

To investigate brain lateralization in ASD, Messina et al. [73] treated zebrafish embryos for 48 h with 1 µM of VPA starting 8 h post fertilization. VPA-treated zebrafish exhibited impaired social behavior and defects in social visual laterality to the image in the mirror [73]. These behaviors corresponded with altered brain asymmetric gene expression and morphology in the thalamus and the telencephalon. Changes in cortical synaptogenesis, synaptic function, behavior, and gene expression in the marmoset (a new world monkey) model of VPA-induced ASD-like phenotypes were studied by Watanabe et al. [71]. In the study, 200 mg VPA was orally administered to pregnant marmosets from GD 60 to GD 66. Whole cell electrophysiological recordings showed altered synaptic plasticity, and microarray brain gene expression studies revealed 1037 differentially expressed genes that are positively correlated to brain gene expression in ASD in humans [71]. These alterations were accompanied by altered infant and juvenile vocalizations as tested by a pulse code modulation (PCM) audio recorder [71].

Polychlorinated biphenyls (PCB), an endocrine disrupting chemical (EDCs), is an environmental contaminant that may affect many neuroendocrine functions [13]. Human epidemiological studies have associated prenatal and early postnatal exposure to high levels of PCBs with an increased risk of ASD [77]. Bisphenol A exposure to Sprague Dawley rats in utero or early postnatal life is associated with an increased risk of ASD-like behavior, manifested as altered social behavior in the partition test and three-chamber test, increased anxiety exhibited on elevated plus maze test, sociosexual preferences recorded from the ultrasonic vocalizations (USVs) during social contact with the opposite sex [78], and social-context deficits when tested in a two-chamber partition paradigm [70]. Rats administered 25 mg/kg of PCB from GD 3 to parturition exhibited significant impairment of social recognition in a two-chamber social recognition paradigm [70]. Isolation-induced social investigation in the adult offspring was reduced. These asocial phenotypes, typical of ASD, were linked to the observed significant reduction in the periventricular nucleus, part of the hypothalamus that mediates social behavior and stress [70].

Prenatal exposure to the organophosphate pesticide chlorpyrifos (CPF) in humans is also associated with impaired social preference, restricted or repetitive behavior, and alteration in social communication [33,72]. Young people are known to be more susceptible to the toxicity of CPF in communities where it is employed for agricultural purposes. Depending on the dose, CPF alters expression levels of genes involved in the development of neuronal communication, motor coordination, and learning (Lan et al., 2017). Several studies used CPF-induced mice model to mimic human ASD symptoms to advance understanding of the condition [79,80,81]. Treatment of pregnant Wistar rats on GD 12.5 with CPF induced in the offspring a significantly decreased number of calls and high latency to start calling in USVs recordings [82]. The type of calls and peak frequencies were not changed in comparison to the control.

To test for ASD-like social and repetitive behavior in mouse model, Lan et al. [72] administered 5 mg of CPF orally to pregnant mice from GDs 12–15. Social preference and social conditioned place preference, social novelty, object recognition, and restricted interest tests were assayed on the offspring at postnatal day 90. CPF-exposed mice showed dampened preference for unfamiliar conspecific and reduced social conditioned place preference with enhanced restricted interest [72].

## 4. Genetic Models in Rodents for ASD-like Behaviors

The heritability of ASD has been calculated as very high, based on twin studies [83,84]. It seems to involve the influence of multiple genes, making it unlikely that a single gene will elucidate the genetics behind the majority of ASD cases. About 15–25% of ASD cases are syndromic, wherein the autistic presentation is just one part of a broader neurological syndrome. The rest of the cases represent non-syndromic ASD, where the main symptoms are communication and social impairment accompanied by stereotyped behaviors [85]. It is accepted that about 10–20% of ASD cases are related to defined rare mutations, genetic syndromes with highly penetrant chromosomal abnormalities, and de novo copy number [86,87] (Table 3).

De novo mutations (DNMs) and risk genes of ASD have been identified among ASD cases and are considered important factors that contribute to the diversity of symptoms, disease severity, and sex-related differences in higher male vs. female genetic liability, susceptibility, and development of ASD in either sporadic or familial pattern [88,89]. These DNMs corresponded with the genes that are mostly associated with biological pathways related to chromatin remodeling, transcriptional regulation, and synaptic functions [90].

Using the homologous recombination and CRISPR/Cas9 technique, numerous models of knock-out/knock-in mice were generated based on the various defined DNMs and potential risk genes of ASD in human patients [91,92,93,94].

The Simons Foundation Autism Research Initiative (SFARI) gene database Mouse Models module provides an integrated envelopment of the current findings at the molecular, cellular, and behavioral levels in ASD (https://gene.sfari.org/database/animal-models/genetic-animal-models/, access online: 27 September 24), extracted from peer-reviewed scientific literature and annotated by expert biologists [95]. The animal models presented in SFARI Gene include thorough descriptions of genetic constructs (such as knockouts, knock-ins, knockdowns, overexpression, and conditional models) as well as a diverse range of phenotypic features documented in scientific studies. Up to publication of this paper, it included a list of more than 252 genetic mice models, 6 copy number variation (CNV) mouse models, 45 induced mouse models, and 8 inbred mouse models; most of these models represent an ASD phenotype relevant to the clinical presentation of autism in humans. 

The mouse genetic models may be classified into several categories according to the type of genetic changes: modeling of autism associated with defined genetic syndromes due to mutations in a single gene, such as fragile X and Rett syndromes. Similarly, non-syndromic autism associated with pathological mutations in single genes, such as the neuroligin or SHANK family genes, and CNVs associated with autism such as 15q11-q13, 16p11.2, and 22q11.2 have been described [96]. Generally, almost all reported studies on mouse models with known ASD risk genes and mutations described that mouse with specific mutations or deletions demonstrated good face validity of autistic-like behavior from the mild phenotype to severe behavioral impairments [91,92,93,94]. Several studies have also reported that genetic models demonstrated predictive validity, with insight into the development of therapeutic approaches to the prevention and treatment of ASD [97,98].

We will bring only the more common examples of genetic animal models that recapitulate human ASD-like behavior. The following are the more common animal models of ASD (summarized in Table 3).

**Table 3:** Common genetic animal models of ASD.

Gene	Conditional Animal Models	ASD-like Phenotype	Autor (Ref.)
**X-linked Methyl CpG Binding Protein 2, *MeCP2***			
*Mecp2^tm1.1Bird^* Mice *Mecp2^tm1.1Jae^*	The targeted deletion that removes exons 3 and 4 of the *Mecp2* gene, resulting in a complete lack of MECP2 protein product. *Mecp2^tm1.1Jae^* mice created by condition disruption of exon 3 of the Mecp2 gene, resulting in the lack of a functional MECP2 protein.	Male hemizygous *Mecp2*-null mice develop a Rett-like phenotype from the 4 weeks of age with raid regression, and die between 6 and 12 weeks of age. Hind limb clasping, tremors, breathing irregularities, loss of muscle tone, reduced locomotion, reduced brain weight and body weight, are the experience of a rapid phenotypic. Female heterogeneous *Mecp2*-null mice develop the same features at 4–6 months of age and typically live a normal lifespan. Two weeks of treatment with Mirtazapine rescue dendritic arborization and spine density of pyramidal neurons and improve phenotypic score.	Guy et al. [99] Bittalo et al. [100] Flores Gutiérrez et al. [97] Chen et al. [101]
Mice *Mecp2-^308/y^*	Stop codon at amino acid position 308, leading to the production of a truncated MeCP2 protein that lacks the C-terminal domain.	Displaying a milder RTT phenotype, a delayed onset of symptoms, and an extended lifespan, due to the presence of partially functional truncated protein.	Shahbazian et al. [102]
Rats *Mecp2^308^*	Expressing a truncated allele of *Mecp2*.	Displayed RTT phenotype: growth retardation, reduced locomotion, impaired social behavior, breathing abnormalities, excessive spontaneous firing activity of neurons in the locus coeruleus.	Wu et al. [103]
*Viaat-Mecp2* ^−/y^	Male *Viaat-Mecp2^−/y^* mice are nearly absent MeCP2 protein from >90% of GABAergic neurons.	Male *Viaat-Mecp2−/y* mice developed RTT and ASD-like phenotype from 5 weeks of age: Motor dysfunction. Repetitive behaviors. Impaired working memory. Reduced levels of Gad1 and Gad2. Decreased GABA immunostaining in the cortex and striatum.	Chao et al. [104]
*Mecp2lox-Stop/Y*	Male *Mecp2* null mice, with genetically restored *Mecp2* expression in targeted GABAergic neurons,	Rescue MECP2 functions. Ablation of RTT phenotype.	Ure et al. [105]
**X-Linked Mental Retardation FMR1 gene, *FMR1***			
Fmr1 KO mice and rats	Loss-of-function models; disruption knockout (KO) of the *FMR1* gene homolog.	Displayed FXS phenotype: Altered social interaction and social play. Social anxiety. Defects in visual attention. Auditory dysfunctions. Cognitive deficits. Repetitive behaviors,. Hyperactivity. Differences in dendritic spines.	Baker et al. [106] Ding et al. [107] Albert et al. [108] Hamilton et al. [109] Barić et al. [110] Curnow et al. [111]
**SH and multiple ankyrin repeat domains proteins (SHANK)**			
Shank3+/- mice	Deletion of the ankyrin repeat region of the Shank3 gene resulted in a lack of full-length SHANK3 protein.	Heterozygous (Shank3+/-) and homozygous (Shank3-/-) showed normal brain anatomic structure and displayed normal developmental trajectory, normal social interaction, normal spatial learning, and repetitive self-grooming in males. Reduced number of USVs. Decreased GLUR1 and AMPA receptor immunoreactivity. Altered LTP in hippocampal CA1 neurons.	Bozdagi et al. [112] Yang et al. [113]
Shank3^e4–9^ mice	Deletion of the Shank3 gene on exons 4–9 produced transcripts of truncated SHANK3 proteins	Homozygous Shank3^e4–9^ mice showed abnormal social communication, decreased novel object preference, impaired spatial learning and memory, increased stereotypic self-grooming, increased number of USV, and affected fine motor coordination. Reduction in brain levels of Shank3-interacting protein Homer1b/c, GKAP, and GluA1.	Wang et al. [114]
Shank3A^−/−^ null mice Shank3B^−/−^ null mice	Targeting the ankyrin repeat domain, resulting in the loss of the longest Shank3α isoform. Targeting the fragment-encoding exons 13 to 16 of the PDZ domain, which led to the complete deletion of both Shank3α and Shank3β isoforms, as well as a reduction in the Shank3γ isoform.	Shank3B−/− mice exhibited a more pronounced ASD-like phenotype, than Shank3A^-/-^ mice: anxiety-like behavior, repetitive self-injurious grooming. Shank3B^−/−^ mice demonstrated impaired social interaction preference for social novelty. Shank3A^−/−^ mice preserved normal social communication, deficit for social novelty recognition, striatal hypertrophy, increased neuronal complexity, and dendritic arbors. Reduced frequency mEPSCs in striatal medium spiny neurons. Reduced protein levels of glutamate receptor subunits GluR2, NR2A, and NR2B.	Peça et al. [115]
Shank3^+^/^ΔC^ mice	Conditional deletion of exon 21 in the C-terminal of the *Shank3* gene, which leads to the expression of a truncated SHANK3 protein.	Only male Shank3+/^ΔC^ mice developed ASD-like phenotype. Decreased level of histone acetylation. Subchronic treatment with romidepsin, class I HDAC inhibitor, transiently rescued social deficits in Shank3+/^ΔC^ mice, elevated the transcriptional level of HDAC2 in PFC, restored β-catenin and restored NMDAR, and elevated expression of actin regulatory genes Grin2. Single I.V. injection with TAT-p-cofilin peptide rescues behavioral deficits and restores NMDAR function. Treatment with UNC0642 inhibitor of EHMT1 and EHMT2, reduced the elevated level of H3K9me2 in the PFC of Shank3+/^ΔC^ mice and rescued autism-like social deficits, and restored NMDAR function	Qin et al. [116] Duffney et al. [117] Wang et al. [118].
**Neuroligin genes, *NLGN***			
Knock-in mice Nlgn1 P89L mice	Knock-in mice with the novel missense mutation P89L in the NLGN1 gene.	Heterozygous Nlgn1 P89L mice: Affected sociability and social dominance. Impaired spatial memory Homozygous Nlgn1 P89L developed a milder ASD-like phenotype: Less impairment in sociability and spatial memory. Either homozygous or heterozygous *Nlgn1* P89L mice demonstrated normal odor discrimination, object recognition, general locomotor activity, stereotypic repetitive behavior anxiety-like behavior, and altered stress-induced USVs. Decreased levels of NLGN1 protein in the brain.	Nakanishi et al. [119]
NL1 KO mice	NLGN1 depletion.	NL1 KO mice exhibited mild deficits in social behavior, impaired spatial memory evaluated by MWM test, and increased repetitive grooming behavior. Impaired hippocampal long-term potentiation. Decrease in the NMDA/AMPA ratio in synapses. A single administration of the NMDA receptor partial coagonist d-cycloserine abolished abnormal grooming phenotype in adult NL1 KO mice.	Blundell et al. [120]
*R215H-Nlgn2* knock-in mice	Mice carrying the R215H mutation in the *Nlgn2* gene lost NLGN protein expression.	*R215H-Nlgn2* mice have growth retardation and demonstrated anxiety-like behavior, impaired spatial learning and memory, and enhanced Startle reflex.	Chen et al. [121]
*R451C-Nlgn*3 knock-in mice	Insertion of *R451C* mutations in an extracellular domain of the *Nlgn*3 gene caused partial retention of NLGN protein in the ER and further proteasomal degradation.	*R451C-Nlgn*3 mutant mice demonstrated controversial phenotype. Tabuchi et al. [122] reported reduced sociability facilitated spatial learning and memory increase in inhibitory synaptic transmission, elevating the inhibition-to-excitation (I/E) ratio of synaptic inputs to cerebellar Purkinje cells. Chadman et al. [123] reported normal reciprocal social interactions, learning, and memory in MWT, similar to WT controls, but demonstrated some delay in the early postnatal developmental trajectory.	Tabuchi et al. [124] Lai et al. [125] Chadman et al. [123].
*Nlgn*3-KO mouse line	*Nlgn*3-KO knockout mouse line with completely depleted NLGN3.	*Nlgn3*-KO mice demonstrated increased decreased social recognition and social novelty preference. Impaired olfaction. Reduced number of USVs in males.	Radyushkin et al. [122]
*Nlgn4*-KO mice	*Nlgn4*-knockout mouse line with chimeric nonfunctional NLGN4 protein.	*Nlgn4*-KO mouse developed abnormality in reciprocal social interactions and communication, decreased USVs, and reduced total brain volume.	Jamain et al. [126]
**Inbred model of idiopathic ASD: BTBR mice**			
*BTBR T+ Itpr3tf/J*	Carries mutations in genes including (nonagouti; Black and Tan), Itpr3tf (inositol 1,4,5-triphosphate receptor 3; tufted), and T (brachyury).	BTBR mice demonstrated natural traits of the core autism symptom: decreased social interaction, increased USVs and abnormal patterns of sonograms, repetitive grooming, lack of corpus callosum and hippocampal commissure, decreased cortical thickness, and thalamic gray matter volume.	Scattoni et al. [127] McFarlane et al. [128] Scattoni et al. [129] Wöhr et al. [130] Meyza et al. [131] Dodero et al. [132]
**Genetic models in nonhuman primates (NHP)**			
*Mecp2* transgenic MF	Mutant MF expressed human *Mecp2* via lentiviral infection of monkey oocytes mitigating MECP2 duplication syndrome.	*Mecp2* transgenic MF exhibited repetitive circular locomotion, increased stress response, reduced social interaction, mildly impaired cognition, significant enrichment in gaba-related signaling pathways, reduced β-synchronization in fronto-parieto-occipital networks EEG studies, and hyperconnectivity in prefrontal and cingulate networks.	Liu et al. [133] Cai et al. [134]
*Mecp2* transgenic Rhesus and cynomolgus monkeys	*Mecp2* mutagenesis was induced by microinjection of *Mecp2*- exon 3-targeted TALEN plasmids into rhesus and cynomolgus zygotes, leading to MECP2 altered expression or function.	Male mutant monkeys were embryonic lethal. Female *Mecp2* mutant monkeys demonstrated stereotypical behaviors, impaired active social interaction, reduced exploration, and affected sleep patterns.	Liu et al. [135] Chen et al. [136]
*Shank3*-deficient MF	CRISPR-Cas9-targeting exon 21 of SHANK3 *in Macaca fascicularis* resulting in expression of non-functional SHANK3 protein	SHANK3-deficient MF capitulated most symptoms of Phelan–McDermid syndrome: Impaired sleep and motor functions. Increased repetitive behaviors. MRI: Abnormal brain global connectivity.	Zhou et al. [137]

### 4.1. Animals with Single Gene Mutations

#### 4.1.1. X-Linked Methyl CpG Binding Protein 2 (MECP2) Gene

Rett Syndrome (RTT) is a neurodevelopmental disorder manifested predominantly in females and is marked by a period of developmental regression typically occurring between 6 and 18 months of age. Most boys with these mutations do not survive to birth or die shortly after birth. The disorder is characterized by cognitive impairments, poor expressive and receptive language, motor dysfunction, stereotypic hand movements, autonomic nervous system irregularities, epilepsy, scoliosis, and behaviors resembling those seen in autism [104,138].

More than 80% of classic RTT patients have mutations in the *Mecp2* gene, which is located on Xq28, encodes the methyl CPG binding protein 2, and is defined as the genetic cause of Rett and ASD syndrome [139,140,141]. Mutations of the *Mecp2* gene are generally sporadic (de novo) including missense mutations, nonsense mutations, and entire exon deletions [142].

MECP2 is an epigenetic factor that is involved in transcriptional tuning of gene repression and activation processes [143], RNA splicing [144], chromatin remodeling [145], and maintenance of DNA methylation pattern [146]. The *Mecp2* gene is expressed in two isoforms with different lengths that are involved in genes’ transcription in neuronal cells [147].

Many mutant mouse and rat models have been developed with relatively high face validity for the behavioral signs associated with Rett Syndrome [99,101,103,148,149], construct validity for morphological and functional changes in brain neuronal functions, and even predictive validity for using pharmacological agents and for wild-type protein gene therapy that rescue neuronal functions [97,98].

Two conditional *Mecp2* knock out (KOs) mice lines, *Mecp2^tm1.1Bird^* which completely lacks MECP2 protein product [99] and *Mecp2^tm1.1Jae^* which expresses small nonfunctional MECP2 protein fragments [101], are the first two generated mouse RETT disease models. The *Mecp2 KOs* mice have quite good face and construct validity, recapitulating many of the behavioral and neuroanatomical phenotypes associated with RTT patients [101,150]. *Mecp2^tm1.1Bird^* mice, lacking MECP2 proteins, exhibited a severe phenotype and short lifespan. Hemizygous male *Mecp2-null* mice are phenotypically normal until 4 weeks of age when they develop a Rett-like phenotype consisting of hind limb clasping, tremors, breathing irregularities, loss of muscle tone, and reduced locomotion. These mice also display a reduced brain weight and body weight, experience a rapid phenotypic regression, and die between 6 and 12 weeks of age. Female mice heterogeneous for *Mecp2* deletions develop the same features later, only at 4–6 months of age and typically live a normal lifespan. Interestingly, hemizygous male MECP2-null mice exhibit a more severe phenotype compared to their female counterparts. Male mice often develop significant symptoms early at 4 weeks, while female mice, who are also hemizygous due to X-chromosome inactivation, typically have milder motor impairments that appear later, after 10 weeks of age.

One of the most consistent changes found in neuronal morphology in animal models of the disorder and postmortem brains of RTT patients are alterations in dendritic and synaptic spine structure indicating impaired synaptic function. Interestingly, in male *Mecp2^tm1.1Bird^* null mice, 2 weeks of treatment with Mirtazapine rescued dendritic arborization and spine density of pyramidal neurons and improved phenotypic score [100]. Long-term treatment with Mirtazapine, a noradrenergic and specific-serotonergic antidepressant, alleviated the RTT phenotype severity and delayed disease progression in adult female heterozygous *Mecp2^tm1.1Bird^* mice [97].

Shahbazian et al. developed a *Mecp2-^308/y^* mouse model that bears a mutation leading to the expression of a truncated protein at amino acid 308, thus preserving the methyl binding domain and a portion of the transcriptional repressor domain of the MECP2 protein [102]. *The Mecp2-^308/y^* mice demonstrated face validity by displaying a milder RTT phenotype, a delayed onset of symptoms, and an extended lifespan, due to the presence of partially functional truncated protein. It was observed that about 10% of RTT patients, with C-terminal deletions of the MeCP2 gene exhibited less severe symptoms [151]. Thus, *Mecp2-^308/y^* mice closely resemble the clinical symptoms observed in this subset of RTT patients.

Rats expressing a truncated allele of *Mecp2* (*Mecp2308*) also reproduce face validity of RTT [103]. *Mecp2308*-null rats displayed growth retardation, reduced locomotion, impaired social behavior, breathing abnormalities, and excessive spontaneous firing activity of neurons in the locus coeruleus [103].

Further, genetic manipulations were employed to precisely target the expression of mutated *Mecp2* genes to specific regions or neuronal subpopulations in the mouse brain and to develop rescue strategies. Thus, Chao et al. demonstrated that Viaat-Cre mice (*Viaat-Mecp2−/y*) with limited Cre expression to GABAergic and glycinergic neurons, and lacking nearly 90% of *Mecp2* from forebrain GABA (γ-aminobutyric acid)-releasing neurons, developed strong face and construct validity recapitulating many RTT and autistic features, including progressive motor dysfunction, repetitive behaviors, and impaired working memory [104]. These findings were accompanied by presynaptic reduction in glutamic acid decarboxylase 1 (Gad1) and glutamic acid decarboxylase 2 (Gad2) levels, and a decrease in GABA immunostaining in cortical neurons and striatal medium spiny neurons.

Later, Ure et al. re-expressed *Mecp2* by crossbreeding male Viaat-Cre mice with females carrying a Mecp2 allele with a floxed STOP cassette between the second and third exons (*Mecp2*lox-Stop/Y) [105]. Male *Mecp2*-null mice, with genetically restored *Mecp2* expression in targeted GABAergic neurons, demonstrated prolonged lifespan, and improvement in signs of ataxia, social abnormalities, and enhanced inhibitory signaling. The improvement was also observed in heterozygous female *Mecp2^+/-^* mice. These findings implemented the regulatory role of GABAergic neurons in RTT etiology and to development of effective therapeutic approaches to cure the Rett syndrome and rescue Mecp2 functions [105].

#### 4.1.2. X-Linked Mental Retardation FMR1 Gene (Fragile X Syndrome, FMR1)

Fragile X syndrome (FXS) represents the most common monogenic form of ASD primarily affecting males and has phenotypic overlap with ASDs. FXS associated with an instability of a trinucleotide CGG repeat expansion within the 5′ untranslated region (5′UTR) of the *Fmr1* gene resulting in the loss of the Fragile X Mental Retardation Protein (FMRP) [152]. In patients with FXS, having more than 200 CGG repeats causes the *Fmr1* gene to become hypermethylated, which silences its expression [153]. FMR1 protein is an RNA-binding protein that regulates protein synthesis-dependent synaptic plasticity [154]. FMRP is present in the brain on proximal dendrites and axons of neuronal cell bodies and mainly associated with polyribosomes [155,156,157].

Mutant *Fmr1* KO mice and rats were generated and displayed altered social interaction and social play behavior, social anxiety, defects in visual attention and auditory dysfunctions, cognitive deficits, repetitive behaviors, and hyperactivity mimicking FXS in humans demonstrating the phenotypic (face) validity with ASD [106,107,109] and were wildly reviewed [111,153]. Many studies have reported construct validity with differences in dendritic spines in *Fmr1*-knockouts [158,159]. However, evaluation of ASD-like behaviors in heterozygous *Fmr*-KO female mice found abnormal sociability in infancy and juvenile age [160,161]. Follow-up observations in adulthood revealed that these abnormal behaviors of *Fmr1*-KO female mice had disappeared, demonstrating the temporal pattern of autistic-like behavior [160].

#### 4.1.3. SH and Multiple Ankyrin Repeat Domains Proteins (SHANK)

Genes of the SH3 and multiple ankyrin repeat domains (*SHANK*) family encode postsynaptic scaffolding proteins present at the postsynaptic densities of excitatory receptors such as AMPA, mGlu, and NMDA glutamate receptors, as well as cytoskeletal proteins, and can also bind to neuroligins [162,163]. Shank is involved in the regulation of the structural organization of excitatory synapses and the stabilization of dendritic spines. Generally, all three SHANK/ProSAP family proteins (SHANK1, SHANK2, SHANK3) have strong genetic evidence of association with ASD [164,165,166,167]. Mutations or deletions in *Shank*3 have been linked to ASD, especially with 22q13.3 deletion syndrome also known as the Phelan–McDermid syndrome (PMDS), which results from *Shank*3 haploinsufficiency or heterozygous *Shank*3 missense variants [166,167,168,169,170]. Individuals with Phelan–McDermid syndrome typically present with global developmental delay, intellectual disability ranging from mild to severe, delayed, or absent speech, and various physical abnormalities. Other common features may include hypotonia, seizures, feeding difficulties, and behavioral issues such as hyperactivity and impulsivity [171].

Researchers have developed multiple strains of mice lacking the *Shank3* gene, that partially recapitulate the general features of PMDS and have been used to explore the role of SHANK3 in the neurological aspects of ASD [112]. All these models have the face and construct validity that developed mild ASD-like phenotype and do not fully recapitulate what is seen in humans.

Bozdagi et al. created mice lacking the full-length *Shank*3 protein, through deletion of the ankyrin repeat region of the *Shank*3 gene [112]. Heterozygous (*Shank3+/*-) and homozygous (*Shank3^-/-^*) animals showed normal brain anatomic structure and displayed normal developmental trajectory [112,113], normal social interaction in the three-chamber test, and normal spatial learning in the Morris water maze [112,113]. The increase in the levels of repetitive self-grooming was gender specific and was observed only in heterozygous (*Shank3^+/-^*) or homozygous (*Shank3^-/-^*) male mice, but not in females. Male heterozygous mice (*Shank3^+/-^*) also showed reduced sniffing time and a reduced number of emitted ultrasonic vocalizations in reciprocal social interaction with the estrus female [112]. Impairment in the novel object recognition test was observed only in homozygous (*Shank3^-/-^*) male mice. In addition, decreased GluR1 and AMPA receptor immunoreactivity were observed and accompanied by impairments in excitatory synaptic transmission and plasticity, in long-term potentiation (LTP) but not long-term depression (LTD) in hippocampal CA1 neurons.

Wang et al. demonstrated that homozygous *Shank3^e4–9^* mice with deletion of the *Shank3* gene on exons 4–9 produced transcripts of truncated SHANK3 proteins [114].

Homozygous *Shank3^e4–9^* mice showed abnormal social communication, decreased novel object preference, impaired spatial learning and memory in the Morris water maze, and increased stereotypic behavior as self-grooming [114]. *Shank3^e4–9^* adult males emitted an increased number of USV calls whereas *Shank3^e4–9^* females emitted fewer calls, and their sonogram represented fewer types of complex calls in comparison to Shank*3^+/+^* mice. Fine motor coordination was affected in *Shank3^e4–9^* mice and male Shank3^e4–9^ mice showed more severe difficulties than female *Shank3^e4–9^*. It was shown that brain levels of Shank3-interacting protein Homer1b/c, GKAP, and GluA1 were reduced in the postsynaptic density in *Shank3^e4–9^* mice [114].

Peça et al. generated two types of *Shank3*-deficient mice [115]. The first type, *Shank3A^−/−^* mice, were created by targeting the ankyrin repeat domain, resulting in the loss of the longest Shank3α isoform. The second type, *Shank3B^−/−^* null mice, involved targeting the fragment encoding exons 13 to 16 of the PDZ domain, which led to the complete deletion of both Shank3α and Shank3β isoforms, as well as a reduction in the Shank3γ isoform [115]. *Shank3B^−/−^* mice exhibited a more pronounced ASD-like phenotype, than *Shank3A^−/−^* mice, characterized by anxiety-like behavior in elevated zero maze test, and repetitive behavior such as obsessive self-injurious grooming [115]. *Shank3B^−/−^* mice demonstrated impaired social interaction and preference for social novelty, whereas *Shank3A^−/−^* mice preserved normal social communication and showed a deficit for social novelty recognition. The findings from this research demonstrated how various *Shank*3 mutations, and the transcription of different SHANK3 protein isoforms, may underlie the heterogeneity of the ASD phenotype, ranging from moderate symptoms to severe impairments. The abnormal behavior observed in *Shank3B^−/−^* mice was accompanied by significant striatal hypertrophy, increased neuronal complexity and dendritic arbors, reduced frequency of mEPSCs in striatal medium spiny neurons, and reduced protein levels of glutamate receptor subunits GluR2, NR2A, and NR2B [115].

Qin et al. further investigated the histone acetylation level in C-terminal (exon 21) heterozygous *Shank3+/^ΔC^* mice, with the deletion of full-length Shank3 protein and in *Shank3^e4–9^* mice [116]. Only male *Shank3+/^ΔC^* mice developed ASD-like phenotype. Qin find an abnormally decreased level of histone acetylation, due to HDAC2 upregulation in the prefrontal cortex (PFC) [116]. Subchronic treatment with romidepsin, class I histone deacetylase (HDAC) inhibitor, transiently rescued social deficits in *Shank3^+/ΔC^* mice, elevated the transcriptional level of HDAC2 in PFC, restored β-catenin and restored NMDAR, elevated expression of actin regulatory genes Grin2. Duffney [117] demonstrated that a single IV injection with TAT-p-cofilin peptide rescues behavioral deficits and restores NMDAR function in *Shank3^+/ΔC^* mice [117]. Wang et al. found that histone methyltransferases EHMT1 and EHMT2, as well as histone lysine 9 dimethylation (specifically catalyzed by EHMT1/2), and level of H3K9me2 were selectively increased in the prefrontal cortex (PFC) of *Shank3+/^ΔC^* mice and autistic human postmortem brains (Brodmann’s area 9) [118,172]. Treatment with UNC0642 (1 mg/kg, i.p., once daily for 3 days), a selective and brain-permeable inhibitor of EHMT1 and EHMT2, reduced the elevated level of H3K9me2 in the PFC of *Shank3^+/ΔC^* mice and rescues autism-like social deficits and restores NMDAR function in *Shank3*-deficient mice similarly to above-described studies [118].

#### 4.1.4. Neuroligin Genes (NLGNs)

A rare mutation of two X-linked neuroligin genes, neuroligin 3 (NLGN3) and neuroligin 4X (NLGN4X) are most prevalent in non-syndromic X-linked ASD and intellectual disability (ID) [173,174,175,176]. The neuroligin (NLGN) family includes five genes, NLGN1, 2, 3, 4X, and 4Y, in humans and only four members, NLGN1, 2, 3, and 4-like in rodents. NLGN3 and NLGN4 genes are X-chromosome-linked genes, while NLGN4Y is an NLGN4 equivalent located on the Y-chromosome. NLGN are cell adhesion molecules that are abundant in the postsynaptic membrane and are responsible for proper synaptic integrity and function rather than synaptic formation and development [177].

Most identified missense mutations are found in the extracellular domain of the proteins and provoke either loss-of-function or gain-of-function of coded proteins [178]. Numerous NLGN loss-of-function or gain-of-function mutation mouse models were developed and the effect of mutations on mice phenotype was investigated. Generally, almost all NLGN models recapitulated the several main ASD core symptoms and demonstrated face and construct validity for those studies aimed to investigate the mechanism of synaptic transmission, enhancing the role of synaptopathy in the complex etiology of autism.

Generally, mice with *Nlgn1* mutation or depletion developed mild ASD-like phenotype, reflecting only a few ASD core symptoms. Nakanishi et al. generated knock-in mice with the novel missense mutation P89L in the *Nlgn1* gene that demonstrated moderate face and good construct validity [119]. *Nlgn1* P89L mice exhibit several behavioral abnormalities reflecting some ASD-like behavior. Heterozygous *Nlgn1* P89L mice showed affected sociability in the three-chamber test and the social dominance test, and impaired spatial memory in the Morris water maze. However, homozygous *Nlgn1* P89L developed more milder ASD-like phenotype and has less impairment in sociability and spatial memory. Both homozygous or heterozygous *Nlgn1* P89L mice demonstrated similar to WT performance in other behavioral paradigms such as odor discrimination, object recognition, general locomotor activity in open-field, stereotypic repetitive behavior assessed by grooming and the marble burying test, and anxiety-like behavior assessed by the EPM task and stress-induced USVs [119]. The decreased levels of NLGN1 protein were found in the cortex, hippocampus, and cortical synaptosome of homozygous and heterozygous *Nlgn1* P89L mice, while the expression levels of mRNA were unchanged.

Blundel et al. investigated *Nlgn1*-KO mice with NLGN1 depletion [120] and reported that they exhibited mild deficits in social behavior, impaired spatial memory evaluated by MWM test, and increased repetitive grooming behavior [120]. These findings were accompanied by impaired hippocampal long-term potentiation, a decrease in the NMDA/AMPA ratio in corticostriatal and hippocampal synapses, with no changes in total synapse density. Interestingly, single administration of the NMDA receptor partial coagonist d-cycloserine abolished abnormal grooming phenotype in adult *Nlgn1*-KO mice.

Chen et al., generated a line of mice carrying the R215H mutation in the *Nlgn2* gene that caused total loss of protein expression in the homozygous mice brains, and partial expression in heterozygous mice [121]. The R215 variant of the *Nlgn2* gene is one of four rare missense mutations revealed in schizophrenia and ASD patients [179]. Homozygous *R215H-Nlgn2* mice have growth retardation and demonstrated anxiety-like behavior, impaired spatial learning and memory, and enhanced Startle reflex. 

Tabuchi et al. generated *R451C-Nlgn3* knock-in mutant mouse lines by insertion of one of the missense mutations associated with ASD, which occurred in an extracellular domain of the *Nlgn3* gene and caused partial retention of NLGN protein in the endoplasmic reticulum with further proteasomal degradation [124]. *R451C-Nlgn3* mutant mice demonstrated reduced sociability. Interestingly, in MWT, R451C-*Nlgn3* mice show facilitation in spatial learning and memory. In addition, an increase in inhibitory synaptic transmission was reported with no changes in excitatory output, indicating a gain-of-function of R451C-substitution mutation. Later, Lai et al. found that R451C substitution mutation, elevate the inhibition to excitation (I/E) ratio of synaptic inputs to cerebellar Purkinje cells and affected physiological developmental elimination of redundant climbing fiber to Purkinje cells synapses on postnatal day 10–in *R451C*-*Nlgn3*-mutant mice [125]. 

Chadman et al. also generated *R451C-Nlgn3* knock-in mutant mice that did not recapitulate any ASD core symptoms [123]. *R451C-Nlgn3* mice exhibited normal reciprocal social interactions, learning, and memory in MWT, similar to WT controls, but demonstrated some delay in the early postnatal developmental trajectory. 

Radyushkin et al. investigated *Nlgn3*-KO knockout mouse line with completely depleted NLGN3 [122]. *Nlgn3*-KO mice demonstrated increased locomotion in the open field, decreased social recognition and novelty preference in the three-chamber test, and impaired olfaction assayed by the buried food-finding test. *Nlgn3*-KO mice also show a reduced number of USVs, measured in males due to contact with female mice in estrous, mitigating deficits in language acquisition during social communication [122].

*Nlgn4*-KO knockout mouse line with depleted functional NLGN4 was generated to assess the effect of the NLGN4 deficit on mice behavior phenotype [126]. *Nlgn4*-KO knockout mice developed prominent selective abnormality in reciprocal social interactions and communication concomitant with a decrease in total brain and cerebellum volume and brainstem MRI volumetric measurements. 

#### 4.1.5. Inbred Model of Idiopathic ASD: BTBR Mice

The *BTBR T+ Itpr3tf/J* strain, arising from the inbred BTBR (Black and Tan Brachyury) strain, carries mutations in genes including (nonagouti; Black and Tan), Itpr3tf (inositol 1,4,5-triphosphate receptor 3; tufted), and T (Brachyury). It stands out as one of the most used animal models due to its natural traits of the core autism symptom for investigating idiopathic ASD and have good face and construct validity [39,127,128,180,181,182]. BTBR mice demonstrated decreased social interaction [127,128] either at an early juvenile age or adulthood, increased USVs and abnormal patterns of sonograms indicating communication deficits between mother and pups [129,130], and repetitive grooming behavior [128,182]. At the neuroanatomic level, this strain shows construct validity with lack of corpus callosum and of hippocampal commissure, decreased cortical thickness, and thalamic gray matter volume [131,132]. In autistic individuals, the analysis of diffusion tensor imaging (DTI) and MRI also demonstrated corpus callosum abnormalities [183,184].

### 4.2. Genetic Models in Nonhuman Primates (NHP)

The development of advanced technologies in gene editing allowed the induction of genes related to ASD in nonhuman primates (NHP), such as cynomolgus monkeys (Macaca fascicularis) [133,136]. Generated transgenic monkey models provide a better face and construct validity to evaluate ASD-like phenotypes due to being closer to humans than mice. 

Liu at al. created mutant cynomolgus monkeys (Macaca fascicularis, MF), expressing human *Mecp2* using lentiviral infection of monkey oocytes, mitigating MECP2 duplication syndrome [133]. *MECP2* transgenic MF exhibited a higher frequency of repetitive circular locomotion, increased stress response, reduced social interaction, mildly impaired cognitive functions, and stable inheritance of transgenic germlines [133]. Cai et al. demonstrated that whole-genome expression analysis carried out in MECP2-overexpressed TG monkeys revealed significant enrichment in GABA-related signaling pathways [134]. This change was linked to reduced β-synchronization in fronto-parieto-occipital networks EEG studies, which correlated with abnormal locomotive behaviors. Moreover, MECP2-induced hyperconnectivity in prefrontal and cingulate networks has been associated with deficits in reversal learning tasks [134]. TALEN-mediated mutagenesis of *Mecp2* in rhesus and cynomolgus monkeys induced dynamic changes in cortical, subcortical, and white matter volumes in MRI imaging analysis [135,136]. All male mutant monkeys were embryonic lethal. *Mecp2* mutant monkeys demonstrated stereotypical behaviors, impaired active social interaction, reduced exploration, and affected sleep patterns [136].

Zhou et al., using CRISPR-Cas9 technology, generated mutations of *Shank3* in cynomolgus macaques (Macaca fascicularis, CM) that were transmitted to their F1 offspring [137]. Functional magnetic resonance imaging (fMRI) showed the abnormal brain global connectivity patterns in mutant CM, resembling ASD. SHANK3-deficient CM developed phenotype capitulated most symptoms of Phelan–McDermid syndrome, characterized by impaired sleep and motor functions and increased repetitive behaviors [137].

## 5. Discussion

We have presented a variety of studies describing the different models of autistic -like behavior in animals, especially in rodents. The investigators generally used a variety of well-accepted behavioral tests that demonstrated different autistic-like features. In many models, however, only a few of the typical behavioral features were assessed. In none of these studies were the behavioral deviations graded according to their severity, as required for the diagnosis of ASD in human. 

ASD and its degree of severity are diagnosed in humans by the presentation of a minimal number of behavioral changes and graded severity, as defined in DSM5 [9]. Indeed, many of the tests used in humans for diagnosing ASD use a scoring system for the different behaviors with a gradual transition from normal to abnormal scores. The score generally also defines the severity of the symptoms. This is true for the common ASD diagnostic tools such as the Child Autism Rating Scale (CARS), Autism Diagnostic Observation Schedule (ADOS), Autism Diagnostic Interview-Revised (ADI-R), and other diagnostic tools [9].

It may be appropriate that animal models of ASD-like behavior should also demonstrate similar measures to those used in humans. The different tests used for the assessment of behavioral changes in animals, especially in rodents, are indeed adequate to assess all specific and typical ASD-like behaviors. However, ASD-like behavior is a combination of changes in several mandatory behavioral traits at different degrees of severity. It may therefore be important to use enough of behavioral tests in order to depict most of the specific behavioral changes of ASD. Thus, studies using too few tests (i.e., only tests for social interaction and communication or repetitive behaviors and anxiety) may be insufficient for the proper identification of an appropriate model of autistic-like behavior. These studies can be used, however, to define specific traits of autistic-like behavior; i.e., communication difficulties, repetitive behavior, restricted interests, abnormal response to sensory stimuli and others, but not the complete diagnostic set of autistic-like behavior.

To the best of our knowledge, there is no accepted scoring system for the definition of ASD-like behaviors in animal models, and most studies just demonstrate several of the behavioral deviations considered to be typical of ASD-like behavior, without assessing their severity [40,185]. Moreover, especially in the non-genetic ASD-like models, it is expected that the severity of the autistic-like behavioral changes will be different among the offspring of the same treated dam. Hence, an accepted scoring system or at least definition of the severity, similar to that in humans, seems to be mandatory. This is apparently true for all animal models that mimic human neurobehavioral and neuropsychiatric diseases.

We therefore propose the following scoring system:

For abnormal results in behavioral tests assessing social interaction, social communication, and social motivation scoring from 0–3, with 0 being the normal (control values), 1—slight abnormality, 2—moderately abnormal, and 3—severely abnormal. We propose to apply tests that evaluate at least two of the three parameters. Thus, the minimal score on both tests is 0 and the maximal is 6. A minimal total score of 3 will define autistic-like behavior in the social interaction’s subfields.

ASD in human is defined only if there are also changes in at least two non-communicative features like repetitive behavior and restricted interests. Hence, in the animal models, at least one of these behaviors must be abnormal. Similar scores should be used from 0–3 with 2 and 3 defining abnormal behavior.

Other associated features like cognitive impairment (including memory and spatial learning), anxiety, impaired motor coordination or sensory impairment are not mandatory but if tested, one has to use for each test similar scores from 0–3, with normal (0), slightly abnormal (1), moderately abnormal (2), and severely abnormal (3). At least one of these should be abnormal.

To sum up, the minimal score that defines autistic-like behavior in all mandatory domains is 5 and the maximal is 12. If a model does not reach the minimal required score, it defines specific behaviors (i.e., social impairment, restricted interests, anxiety, etc.) but it is not a full model for ASD-like behavior

Defining a score for ASD-like behavior would encourage all investigators to use at least four different behavioral tests to appropriately assess models for ASD-like behavior. Using fewer tests will define individual ASD -like behaviors but are not models for most or all features of ASD-like behavior. It would also enable us to define the animals that present with ASD-like behavior in the litter and carry out the planned specific studies only on those that present the minimal score. Moreover, it will also enable a better evaluation of the possible benefits of preventive and/or therapeutic modalities used in these models.

This is a theoretical scoring system that is proposed by us which has to be tested with a large sample of animals and appropriate sensitivity/specificity statistics.

In our previous studies on the ASD-like behavioral changes induced by the early postnatal administration of VPA, we proposed a composite scoring system for the classification of ASD-like behavioral changes [40]. We combined Z scores of three behavioral parameters: self-grooming frequency in the open field test, percent of incorrect turning in the T maze, and social novelty preference to a familiar stimulus from the three-chamber social interaction test. We indeed found that the autism composite score was higher in the VPA-treated male and female mice in comparison to controls and was normalized by the coadministration of SAMe [40]. However, we did not assess the severity of the recorded behavioral changes which can be calculated for each animal from the extent of differences from controls.

Defining a score for ASD-like behavior would encourage investigators to use sufficient behavioral tests in order to appropriately assess full models for ASD-like behavior. If one uses only a few tests, it is rather advisable to name a model according to the behavioral impairment. For example: animal model of impaired social interaction/communication/social memory to reflect its phenotypical validity so as to avoid artifactual result generalization.

Since a variety of neuropsychiatric disorders are defined by using clinical scores, a scoring system can also be used in animal models of other neuropsychiatric disorders.

## 6. Conclusions

Appropriate animal models of human diseases are of utmost importance for understanding their etiology, pathogenesis, and treatment. While models for diseases that have biological markers are easily defined, non-genetic animal models for neurobehavioral and neuropsychiatric disorders generally lack biological markers. Hence, autistic-like behavior is not easily defined because specific neurobehavioral features in an animal do not exactly replicate human behavior. However, adequate valid behavioral tests have been developed, especially in rodents, to measure behavioral deviations similar to humans. Thus, genetic and environmentally induced models of behavioral deviations similar to those observed in human ASD were developed. They have enabled the study of ASD (ASD-like behavior) from the etiologic, pathogenetic, and therapeutic aspects. Such models mimicking ASD-like behavior exist not only in rodents but also in higher mammals such as primates as well as in lower animals such as zebrafish. However, we should be careful in our neurobehavioral assessments to be sure that the accurate models indeed meet most clinical behavioral manifestations of human ASD. Ideally, we should use a scoring system of behavioral deviations and assess their severity. Such a scoring system is proposed in this review, but still has to be tested for sensitivity and specificity.

## Figures and Tables

**Table 1:** Studies of immune activation in animal models of ASD-like behaviors.

Animals	Testing Paradigm	Phenotype Manifestations	Authors
Pregnant C57BL/6 were treated with 300 μg of IgG on GD 13.5	OF, Y-Maze, marble burying, clock maze	Adult offspring show abnormal sociability, impaired motivation, stereotypic and/or compulsive behavior, learning inflexibility	Brimberg et al. [50]
C57BL/6 dams were injected with multiple synthetic antigenic epitopes before pregnancy; inducing autoimmune response	Open arena social approach, three-chambers, self-grooming, marble burying, USVs, MWM, OF, EPM, light and dark box	Adult offspring manifest reduced number in USVs, repetitive behaviors, diminished interest in social interaction, neurodevelopmental delays	Jones et al. [44]
Sprague Dawley Rat dams received injections of 21 custom synthetic peptides (LDH-A, LDH-B, STIP1, and CRMP1), 4 weeks before pregnancy	Neurodevelopmental test, USVs, EPM, OF, three-chamber, pre-pulse inhibition reciprocal social behavior social novelty test	Adult offspring show impaired social behavior, dampened social reciprocity	Bruce et al. [53]
C57BL/6J dams were injected IP with 20 mg/kg poly(I:C) on GD 12.5	Three-chamber Self-grooming, EPM OF	Adult offspring show declined sociability, social recognition, and anxiety Excessive self-grooming Increased NKCC1 Dendritic spines Reactive microglia in PFC	Zhang et al. [58]
C57BL/6J dams were treated with Poly I:C on GD 12.5 Pups were treated with LPS on PND 9	USVs, scent marking, social recognition, OF, rotarod	ASD-like phenotype more severe in males than in females Altered social behavior Repetitive behaviors Anxiety Altered USVs in both sexes	Carlezon et al. [59]
C57bL/6 dams were IP injected with 15 µg/kg LPS on GD 15	Three-chamber, stereotypic behavior test	Adult offspring show altered social interaction, stereotyped self-grooming, abnormal BDNF and interleukin 17A in the hippocampus and cortex These altered behaviors were absent at age 28	Dutra et al. [60]
Female zebrafish were treated with Poly(I:C) intraperitoneally at 24 h before mating	Three-chamber, shoaling, OF social preference test	Offspring zebrafish show impaired social approach/cohesion, altered villin-1 (vil1) pathway	Wu et al. [61]
C57BL/6J dams were IP injected with 20 mg/kg poly (I:C) on GD 12.5	OF, EPM, grooming test, marble burying Three-chamber test	Adult offspring show reduced locomotion, increased anxiety, higher repetitive digging, higher repetitive stereotyped behavior, impaired social interaction and recognition memory	Zeng et al. [62]
Balb/c dams were exposed to *Mycobacterium tuberculosis* (*Mtb*) via aerosol infection on GD 12.5	Three-chamber test, self-grooming	No deficit in social behavior Increased repetitive self-grooming.	Manjeese et al. [63]
Pregnant rhesus monkey was treated on GDs 30, 44, 58, 72, 86, 100 with IgG from mother of ASD patient	Reciprocal social interaction, three-chambers, MIR	IgG-ASD offspring were asocial to conspecific; showed impaired reciprocal social interaction; had abnormal frontal lobe white matter	Bauman et al. [55]
C57BL/6 dams were IP injected with 75 μg/kg LPS on GD 14.	Three-chamber, OF, EPM, forced swim tail suspension	Impaired social interactions and social recognition Altered locomotion anxiety Depression	Wu et al. [64]

**Table 2:** Studies in animal models of chemically induced-ASD-like behavior.

Animal	Test Paradigm	Phenotype Manifestations	Authors, Ref.
ICR mice were treated with 300 mg/kg VPA on PND 4	Three-chamber, EPM, Water T-maze, OF test	VPA-increased grooming frequency, impaired sociability in males increased anxiety-like behaviors in females.	Ornoy, et al. [40]
Pregnant Wistar rats were administered 600 mg/kg of VPA on GD 12.5	Three-chamber, reciprocal social interaction, OF/self-grooming, and EPM	Decreased social interactions and recognition, increased anxiety and nociceptive threshold	Hirsch et al. [37]
Treating zebrafish embryos with 5, 50, and 500 µM of VPA at 8 h post fertilization	Light and dark swim speed/preference test, larval social test, mirror attack, shoaling, and social contact test	Hyperactive movement disorder and thigmotaxis, reduced social interaction, macrocephaly	Chen et al. [69]
Rats administered 25 mg/kg of PCB from GD 3 to parturition	Two-chamber social paradigm	Impairment of sociability and social recognition	Jolous-Jamshidi et al. [70]
200 mg VPA was orally given to pregnant marmosets from GD 60 to 66	Pulse code modulation (PCM) audio recorder	Altered infant and juvenile vocalizations	Watanabe et al. [71]
5 mg of CPF to pregnant mice from GDs 12–15	Three-chamber, social interaction, object recognition and restricted interest tests	Enhanced restricted interest. reduced social conditioned place preference, dampened social recognition	Lan et al. [72]
Treated zebrafish embryos for 48 h with 1 µM of VPA starting 8 h post fertilization	Mirror test, two-chamber social paradigm	Exhibited impaired social behavior and social visual laterality, with altered brain asymmetry	Messina et al. [73]

## Data Availability

Not applicable.

## Abbreviations

ADI-R	Autism Diagnostic Interview-Revised
ADOS	Autism Diagnostic Observation Schedule
AMPA	α-amino-3-hydroxy-5-methyl-4-isoxazolepropionic acid
ASD	Autism spectrum disorder
BM	Barnes maze
BTBR	Black and Tan Brachyury
CARS	Child Autism Rating Scale
Cas9	CRISPR-associated protein 9
CNV	Copy number variation
CPF	Chlorpyrifos
CRISPR	Clustered regularly interspaced short palindromic repeats
DNM	De novo mutation
DSM-5	Diagnostic and Statistical Manual
DTI	Diffusion tensor imaging
EDC	Endocrine disruptor
EHMT	Euchromatic histone-lysine N-methyltransferase
EPM	Elevated plus maze
FMR1	Fragile X-linked Mental Retardation gene
FMRP	Fragile X Mental Retardation Protein
FXS	Fragile X Syndrome
GABA	γ-aminobutyric acid
Gad	Glutamic acid decarboxylase 1
Gad2	Glutamic acid decarboxylase 2
GD	Gestational day
GKAP	Guanylate kinase-associated protein
GluR	Glutamate receptor subunit
HDAC	Histone deacetylase
IgG	Immunoglobulin G
IL-6	interleukin 6
IV	Intravenous
KO	Knockout
LPS	Lipopolysaccharide
MECP2	Methyl CpG Binding Protein 2
mEPSC	Miniature excitatory postsynaptic current
MIA	Maternal immune activation
MRI	Magnetic resonance imaging
MWM	Morris Water Maze
NLGN	Neuroligin
NMDA	N-methyl-D-aspertate
NOR	Novel Object Recognition
NR2	NMDA receptor subunit
OF	Open Fields
P2Y	Purinergic receptor
PCB	Polychlorinated biphenyls
PFC	Prefrontal cortex
PMDS	Phelan–McDermid syndrome
PND	Post-natal day
PolyIC	Polyinosinic-polycytidylic acid
RNA	Ribonucleic acid
RTT	Rett Syndrome
SFARI	Simons Foundation Autism Research Initiative
SHANK	SH3 And Multiple Ankyrin Repeat Domain
TALEN	Transcription Activator-Like Effector Nuclease
USV	Ultrasonic vocalization
VPA	Valproic acid
WT	Wild type
WTM	Water T-maze
